# Cellular Reprogramming toward the Erythroid Lineage

**DOI:** 10.1155/2011/501464

**Published:** 2011-07-26

**Authors:** Laura J. Norton, Alister P. W. Funnell, Richard C. M. Pearson, Merlin Crossley

**Affiliations:** School of Biotechnology and Biomolecular Sciences, University of New South Wales, Sydney, NSW 2052, Australia

## Abstract

Haemoglobinopathies such as thalassaemia and sickle cell disease present a major health burden. Currently, the main forms of treatment for these diseases are packed red blood cell transfusions and the administration of drugs which act to nonspecifically reactivate the production of foetal haemoglobin. These treatments are ongoing throughout the life of the patient and are associated with a number of risks, such as limitations in available blood for transfusion, infections, iron overload, immune rejection, and side effects associated with the drug treatments. The field of cellular reprogramming has advanced significantly in the last few years and has recently culminated in the successful production of erythrocytes in culture. This paper will discuss cellular reprogramming and its potential relevance to the treatment of haemoglobinopathies.

## 1. Introduction: Globin Gene Regulation and Haemoglobinopathies

The various blood cell lineages in mammals arise from a multipotent haematopoietic stem cell via particular differentiation pathways. One of these pathways, erythropoiesis, leads to the production of red blood cells (RBCs), which transport oxygen and carbon dioxide around the body by means of the intracellular metalloprotein haemoglobin (Hb). Hb is a tetramer, consisting of two *α*-globin and two *β*-globin subunits. In mammals, these globin chains are encoded by two gene loci: the **α*-globin* locus and the **β*-globin *locus. In humans, the **α*-globin* locus consists of the embryonic **ζ**- and adult **α*-globin* genes, and the **β*-globin* locus comprises the embryonic **ε**-, foetal **γ*^G^*- and **γ*^A^-*, and adult **δ*- and *β*-globin* genes [1, 2]. The *globin* genes expressed from these loci differ from embryonic to adult erythropoiesis in order to meet varying oxygen demands and facilitate placental transfer of oxygen from mother to embryo [3]. 

There are a number of severe diseases caused by the disruption of adult *globin* genes, including thalassaemias and certain types of anaemia. According to the World Health Organisation, approximately 5% of the world's population carry genes involved in Hb disorders, and as such, they present an enormous health burden. Thalassaemia is caused by a reduction or abolition of the expression of one or more *globin* genes, resulting in an imbalance of **α*-* and **β*-globin* chains in red blood cells and consequent anaemia [1, 4]. Sickle cell anaemia is another prevalent haemoglobinopathy and is caused by a mutation in the adult **β*-globin* gene which generates a single glutamic acid to valine amino acid substitution. This mutation leads to the polymerisation of globins in venous circulation [5, 6], which can trigger a rigid and sickled cell phenotype [7, 8] and results in a number of acute conditions such as vaso-occlusion, splenic sequestration, and haemolytic anaemia [9]. 

There are currently a number of treatments available for patients suffering from thalassaemia and sickle cell anaemia. The most common is packed red blood cell transfusion, but this is associated with a number of problems, such as sufficiency of supply, bacterial and viral infection, biochemical and biomechanical changes during storage (red blood cell storage lesions), and the risk of immune rejection from the patient [10, 11]. Furthermore, blood transfusions are ongoing throughout a patient's life and often lead to a potentially fatal buildup of iron and associated reduction in organ activity. 

Another potential therapeutic option involves the reactivation of foetal *γ*-globin expression in adult patients. Residual production of foetal **γ*-globin* persists naturally throughout life, and levels vary between individuals [12, 13]. This persistent expression allows two **γ*-globin* chains to combine with two adult **α*-globin* chains to form what is known as foetal Hb (HbF). As only the adult **β*-globin* gene is mutated in sickle cell anaemia, affected infants are protected from severe symptoms until they reach several months of age, due to the large amount of HbF still in circulation at birth [14]. Furthermore, patients who have inherited alleles associated with increased levels of HbF, known as hereditary persistence of foetal Hb (HPFH), are protected into adulthood [15]. Similarly, a more asymptomatic disease phenotype has also been shown in patients with *β*-thalassaemia who exhibit higher levels of HbF [16]. Together, these observations indicate that increased foetal **γ*-globin* is able to compensate in part for the loss of adult **β*-globin* function and thus ameliorates the symptoms of certain adult haemoglobinopathies. Accordingly, a number of drug treatments for *β*-thalassaemia and sickle cell disease, for instance, 5-azacitidine, hydroxyurea, and butyrate, all act by nonspecifically reactivating foetal **γ*-globin* gene expression by various mechanisms. The effects of these drug treatments are transient and thus require ongoing administration. There is evidence that long-term administration of these drugs has chronic side effects, consistent with their lack of specificity [8, 17]. 

As the existing methods of treatment for these haemoglobinopathies remain inadequate, alternative forms of therapy are currently being sought, and stem cell therapies should be considered. This paper will discuss progress in utilising novel cellular reprogramming techniques to treat RBC diseases.

## 2. Cellular Reprogramming

Stem cells, both embryonic and adult, have the ability to differentiate into various cell types, making them a potentially attractive treatment option. Embryonic stem cells (ESCs) and adult stem cells (ASCs) each have their own strengths and disadvantages in these strategies. ESCs are more easily grown in culture and are pluripotent, meaning that they are able to differentiate into any cell of the body. The practicality of widespread ESC use for therapeutic purposes, however, has been questioned due to issues of supply and ethical and legal considerations. Moreover, these cells carry the risk of allogeneic immune rejection. ASCs, on the other hand, overcome some of these problems as they can be harvested from each individual patient. These cells, however, offer a different set of challenges. They are not abundant and are difficult to obtain, often being harboured in internal organs such as the gut and bone marrow. They have proven difficult to culture *in vitro *[18], and furthermore, they are widely believed to be multipotent rather than pluripotent and are thus only able to differentiate into certain cell types [19]. Cellular reprogramming potentially overcomes these issues and may offer new treatment methods for a range of diseases, including those of RBCs.

### 2.1. Classic Cellular Reprogramming

**Figure 1:**
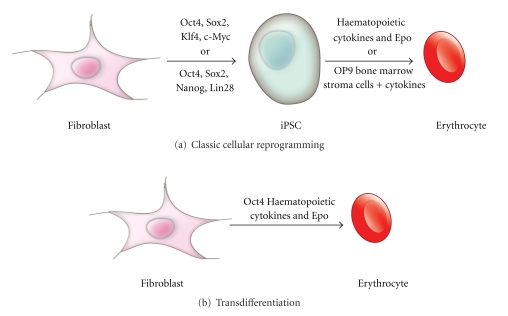
Diagrammatic representation of cellular reprogramming compared to transdifferentiation in the development of erythrocytes. (a) Fibroblasts cells can be reprogrammed into pluripotent cells through the introduction of various exogenous factors. From this induced pluripotent state, these cells can be either cultured with various cytokines supporting erythrocyte differentiation and growth, for instance, IL-3, IL-6, and Erythropoietin (Epo) or cultured with OP9 bone marrow stroma cells to differentiate into erythrocytes. (b) Fibroblasts cells can be reprogrammed directly into erythrocytes through the introduction of Oct4 and culturing with Epo in conjunction with other haematopoietic cytokines.

Takahashi and Yamanaka [20] were the first to show that it is possible to take differentiated somatic cells and transform them into cells with pluripotent potential. They began by identifying a pool of 24 transcription factors which are important in maintaining stem cell traits and used retroviral transduction to express these factors in murine embryonic and adult fibroblasts. They found that these cells then displayed characteristics and properties comparable to those of pluripotent ESCs. They were able to further refine the required transcription factors by setting up numerous combinations to determine which were essential to this process and identified four factors, Oct4, Sox2, Klf4, and c-Myc, needed to transform a somatic cell to an induced pluripotent stem cell (iPSC). Takahashi et al. [21] then applied these four factors to human cells and transformed neonatal and adult human fibroblasts into human iPSCs (hiPSCs). This was a valuable subsequent step as it showed that this process can be reproduced with human cells and could thus potentially be utilised for stem cell-based therapies of human disease. Several other transcription factor combinations have since been shown to be sufficient to reprogram somatic cells [22–24] and one particular combination, involving OCT4, SOX2, NANOG, and LIN28, is particularly efficient and is now widely utilised [25].  Figure 1(a) depicts the process of cellular reprogramming.

There are several potential issues associated with using iPSCs to treat disease. For example, a number of the transcription factors used to generate these cells, including c-Myc, Klf4, and Lin28, are oncogenes, and their misexpression can lead to cancer [26–28]. In order for iPSCs to be differentiated into a cell type of choice, the retrovirally transmitted genes need to be switched off or removed to reduce the possibility of their inducing tumours [22, 29]. Another potential issue is that the transplantation of any cells that have not been fully differentiated from the iPSC state could lead to the formation of cancerous teratomas. Any cells remaining which are still in a pluripotent state could multiply and, without the appropriate growth controls, would have the potential to result in tumours in transplant patients. To potentially avoid the issue of tumour formation and uncontrolled proliferation of iPSCs, an alternative methodology known as transdifferentiation is also being considered as a potential treatment strategy. 

### 2.2. Transdifferentiation

Transdifferentiation is achieved by introducing various exogenous factors into a differentiated cell, such as a fibroblast, to directly convert it into another type of differentiated cell, thereby bypassing the pluripotent state (Figure 1(b)). As early as 1990, Choi et al. [30] were able to convert various cells types, including dermal fibroblasts and chondroblasts, into mononucleated, striated myoblasts that were indistinguishable from normal myoblasts *in vivo*. This was achieved through the expression of the myogenic regulatory factor MyoD, a transcription factor known to be involved in the determination of muscle cells. Shortly after this, another group showed that it was possible to turn myeloid 416B cells into mast cells through the forced expression of GATA-2 and GATA-3, two transcription factors which play important roles in haematopoiesis [31]. Halder et al. [32] investigated the effects of ectopically expressing the gene *eyeless (ey)*, an orthologue of the mammalian *Pax6* gene, in *Drosophila* and found that eye structures formed in places such as the wings and legs. 

Since Takahashi and Yamanaka's pioneering studies in cellular reprogramming, the field of transdifferentiation has advanced considerably. Zhou et al. [33] investigated the effects of expressing Ngn3, Pdx1, and Mafa, transcription factors involved in *β*-cell differentiation, on exocrine cells of the adult pancreas. They found that the coexpression of these factors was able to convert the exocrine cells into *β*-cells. The induced *β*-cells were identical to endogenous *β*-cells in morphology and showed similar expression of genes associated with *β*-cell function. These cells can also rescue the phenotype of hyperglycaemia, as they are able to secrete insulin and remodel surrounding vasculature. In other work, Vierbuchen et al. [34] utilised the neural-specific transcription factors, Ascl1, Brn2, and Myt1l, to rapidly convert both murine embryonic fibroblasts and adult fibroblasts directly into functional neurons. These induced neuronal cells express neuron-specific proteins, generate action potentials, and form functional synapses. A further advance has shown that it is also possible to transdifferentiate fibroblasts into functional neural progenitor cells by transient induction of Oct4, Sox2, Klf4, and c-Myc in cells cultured in a defined neural reprogramming medium [35]. This process bypassed the generation of iPSCs and gave rise to multipotent progenitors with the capacity to expand and differentiate into a number of neural lineages. All of these experiments indicate that it is possible to direct a differentiated cell to another cell fate through the application of extragenic factors.

## 3. Cellular Reprogramming as a Potential Treatment for Anaemia

Advances in cellular reprogramming have raised the attractive possibility that this technology could be utilised to generate a limitless source of immune-matched, pathogen-free erythrocytes for transfusion. Efforts were thus made to produce mature erythroid cells from hiPSCs in culture. An initial study by Feng et al. [28] revealed some practical difficulties. They found that hiPSCs are capable of generating haematopoietic cells with phenotypic and morphological characteristics similar to those derived from hESCs; however, these hiPSC-derived cells exhibited a dramatically reduced capacity (by greater than 1000-fold) to generate erythroid cells. 

A subsequent study by Lapillone et al., however, showed that it was indeed possible to produce significant numbers of mature erythroid cells from hiPSCs *in vitro* [36]. This group employed the methods outlined by Thomson's group [25] using *OCT4, SOX2*,* NANOG,* and *LIN28* to convert fibroblasts to hiPSCs. They then cultured these cells in medium containing the cytokines SCF, TPO, FLT3 ligand, rhu BMP4, rhu VEGF-A165, IL-3, IL-6, and erythropoietin (Epo). These culture conditions were optimised to obtain embryoid bodies that display early erythroid commitment. They analysed the expression profiles of these cells over 20 days of culture and found that pluripotent stem cell markers decreased whilst the erythroid markers CD36, CD235a, and CD71 increased. Cells at day 20 were found to have a high erythroid potential and were plated in sequential cocktails of cytokines comprising SCF, IL-3, and/or Epo. Erythroid maturation was achieved after another 25 days and was confirmed by morphological examination and by flow cytometric analysis of erythroid markers (CD235a^hi^, CD71^hi^, and CD36^lo^). Furthermore, these erythroid cells were able to enucleate, albeit with reduced capacity compared to hESC-derived erythroid cells, and were found to express functional Hb (predominantly HbF). These hiPSC-derived erythroid cells were compared to those differentiated from hESCs, and no significant differences were detected in terms of erythroid commitment, expression of erythroid markers, and type and functionality of Hb. 

The study by Lapillone et al. revealed that while functional RBCs could be generated from hiPSCs, when compared to hESCs, hiPSCs were shown to have reduced (approximately 8-fold) amplification potential in producing mature erythroid cells. A recent study has set out to determine whether this reduced efficiency is an intrinsic property of hiPSCs, or whether it can be increased by altered culture conditions [37]. This study showed not only that it is possible to generate RBCs from hiPSCs with similar efficiency to hESCs, but also that these cells can be differentiated from hiPSCs generated using episomal vectors, obviating the need for potentially deleterious retroviral transfection. This group cultured hiPSCs derived from human fibroblasts with the OP9 bone marrow stroma line to induce hematopoietic differentiation and followed this with selective expansion of erythroid cells in serum-free media with cytokines supporting RBC differentiation. The erythroid cultures established in this study consisted of a pure population of CD235a+ CD45− leukocyte-free RBCs which had robust expansion capabilities, as well as a long lifespan of up to 90 days. These hiPSC-derived cells can enucleate and were shown to express foetal *γ*- and embryonic *ε*-globin demonstrating successful reprogramming of the *β*-globin locus. The results from this study show that it is possible to produce significant numbers of erythroid cells from fibroblast-derived hiPSCs, and that thus cellular reprogramming could contribute to the treatment of haemoglobinopathies. Furthermore, since these RBCs are generated from transgene-free hiPSCs, they circumvent problems associated with genomic integration and undesired reactivation of reprogramming factors. 

Another recent study has shown that it is possible to use transdifferentiation to produce haematopoietic cell lineages, including mature erythrocytes [38]. In this work, Szabo et al. transduced human dermal fibroblasts with *OCT4* alone and showed that these cells then express the panhaematopoietic marker CD45 but lack the pluripotency marker Tra-1-60. They then cultured these cells in a haematopoietic cytokine cocktail containing SCF, G-CSF, FLT3LG, IL-3, IL-6, and BMP-4 supplemented with Epo and observed an emergence of cells expressing adult *β*-globin protein and the red blood cell marker Glycophorin-A. Moreover, these erythroid cells were able to mature in culture and underwent enucleation. In addition, the authors noted that these erythroid cells express adult rather than embryonic globins, unlike cells derived from human pluripotent stem cells [39, 40], perhaps suggesting that they have indeed been transdifferentiated from adult fibroblasts, bypassing the hiPSC stage. As the techniques used in this study avoid the pluripotent state as well as have a high yield, expansion capacity, and clinical feasibility, this strategy could provide a reasonable basis for autologous cell replacement therapies. 

These studies demonstrate that it is possible to differentiate hiPSCs into mature erythrocytes *in vitro* for transfusions. In order to utilise these methods to treat RBC diseases successfully, fibroblasts could potentially be taken from either a healthy immunomatched individual, or a universal donor, and be converted to erythrocytes via the hiPSC state. These cells could then be mass produced in culture and stored for transfusion. A study has already developed hiPSCs from an individual with a Bombay phenotype of the ABO blood group system, where the ABH antigen is not expressed on RBCs, and blood can be donated to anyone [41]. This group has further demonstrated that it is possible to differentiate haematopoietic lineages from these cells although they have not explicitly shown mature erythrocyte differentiation. This possible treatment method circumvents many of the problems associated with current transfusions, such as questionable quality, lack of supply, and immune-rejection. However, issues still remain, such as cost and the risk of iron overloading. 

## 4. Cellular Reprogramming as a Potential Cure for Anaemia

In order to cure, rather than treat, RBC diseases, healthy progenitor cells must be transplanted into patients and subsequently repopulate the haematopoietic system. A promising study has already shown that it is possible to use reprogrammed cells to treat sickle cell anaemia in mice. Hanna et al. [42] took cells from the tail tips of a humanized sickle cell anaemic mouse model, in which the mouse **α*-globin* genes have been replaced with human **α*-globin*, and the mouse **β*-globin* genes have been replaced with human **γ*^A^* and **β*^S^* (sickle) globin genes [43], and through adaptation of the protocol developed by Takahashi and Yamanaka, who reprogrammed these cells into iPSCs. Once this was accomplished, they were able to correct the mutant **β*^S^-*globin gene by replacing it with a copy of the wildtype human **β*-globin* gene through homologous recombination. Following this, the iPSCs were differentiated into haematopoietic progenitors through the ectopic expression of HoxB4, and by growing in haematopoietic cytokines on an OP9 bone marrow stroma cell line. These corrected haematopoietic progenitor cells were transplanted into the sickle mice after irradiation. This group then carried out extensive testing to determine whether the treated mice displayed any further signs of the disease. Firstly, functional correction was evaluated by electrophoresis for the human *β*-globin proteins **γ*^A^* and **β*^S^*, and they found a significant increase in **γ*^A^*- and a decrease in **β*^S^*-globin. Blood counts were also performed 12 weeks after transplant, which showed that treated mice had increased RBCs, Hb, and packed cell volume as well as reduced reticulocytes, a common indicator of sickle cell disease and severity. Lastly, they examined symptomatic indicators of sickle cell anaemia such as urine concentration, body weight, and breathing rate and found that all three of these parameters were ameliorated in the treated mice. This study thus provides an important proof of principle that cellular reprogramming can be employed to correct erythroid disorders, albeit in this case, in conjunction with gene therapy. 

## 5. Conclusions

Normal erythropoiesis is dependent upon the correct expression of *globin* genes. Where *globin* genes are incorrectly expressed, or are mutated, anaemia or thalassemia results. Current therapy for these disorders involves packed red blood cell transfusions, which are limited by supply, risk of infection, expense, and patient rejection. Drug-based therapies involve the nonspecific reactivation of foetal globins and have long-term side effects. In seeking alternative strategies, recent advances have shown that cellular reprogramming can now generate large quantities of red blood cells in culture, potentially for use in transfusions. Furthermore, these strategies have been successfully combined with gene therapy to treat a sickle cell anaemia mouse model, suggesting that cellular reprogramming will provide a realistic future alternative to conventional treatment of haemoglobinopathies.
